# Hemicellulolytic enzymes in lignocellulose processing

**DOI:** 10.1042/EBC20220154

**Published:** 2023-04-18

**Authors:** Heidi Østby, Anikó Várnai

**Affiliations:** Norwegian University of Life Sciences (NMBU), Faculty of Chemistry, Biotechnology and Food Science, P.O. Box 5003, N-1432 Aas, Norway

**Keywords:** biorefinery, enzymatic valorization, hemicellulose, plant biomass

## Abstract

Lignocellulosic biomass is the most abundant source of carbon-based material on a global basis, serving as a raw material for cellulosic fibers, hemicellulosic polymers, platform sugars, and lignin resins or monomers. In nature, the various components of lignocellulose (primarily cellulose, hemicellulose, and lignin) are decomposed by saprophytic fungi and bacteria utilizing specialized enzymes. Enzymes are specific catalysts and can, in many cases, be produced on-site at lignocellulose biorefineries. In addition to reducing the use of often less environmentally friendly chemical processes, the application of such enzymes in lignocellulose processing to obtain a range of specialty products can maximize the use of the feedstock and valorize many of the traditionally underutilized components of lignocellulose, while increasing the economic viability of the biorefinery. While cellulose has a rich history of use in the pulp and paper industries, the hemicellulosic fraction of lignocellulose remains relatively underutilized in modern biorefineries, among other reasons due to the heterogeneous chemical structure of hemicellulose polysaccharides, the composition of which varies significantly according to the feedstock and the choice of pretreatment method and extraction solvent. This paper reviews the potential of hemicellulose in lignocellulose processing with focus on what can be achieved using enzymatic means. In particular, we discuss the various enzyme activities required for complete depolymerization of the primary hemicellulose types found in plant cell walls and for the upgrading of hemicellulosic polymers, oligosaccharides, and pentose sugars derived from hemicellulose depolymerization into a broad spectrum of value-added products.

## Introduction

Lignocellulosic biomass is an abundant source of carbon-based renewable material, consisting of mainly cellulose, hemicellulose, and lignin, and, to a lesser amount, also pectin, proteins, and minerals. Plant-derived biomass is biodegradable and is depolymerized in nature by lignocellulose-degrading fungi and bacteria utilizing a vast array of cellulolytic, hemicellulolytic, and lignin-active enzymes. Application of (a fraction of) these enzymes to convert lignocellulosic biomass into a broad range of products in a biorefinery context is highly appealing for multiple reasons. Enzyme technology complies with the basic principles of green chemistry as enzymes are highly selective catalysts and function under mild process conditions. Furthermore, the future bioeconomy depends on the valorization of local lignocellulosic biomass resources and their transformation into energy carriers and (bio)materials. Finally, lignocellulose-derived materials store carbon dioxide (captured by plants from the atmosphere) temporarily to varying duration, depending on their use.

Traditionally, the longest-utilized part of lignocellulosic biomass is cellulose, in the form of paper, while lignin was long recognized as a by-product. Even today, lignin is often burnt for energy recovery in the forms of electricity and heat in the pulp and paper industry and bioethanol production plants [1]. Over the past century, our continuously growing understanding of the lignocellulose structure in various plants and plant tissues as well as of the lignocellulose-degrading machineries of plant biomass-decomposing organisms together with the urgency of finding sustainable feedstocks alternative to fossil resources have led to the development of an array of bioconversion technologies for biomass refinery and the identification of a broad range of products.

In addition to agricultural by-products, it is noteworthy that hemicelluloses are abundant in waste streams from processing of plant seeds for food production. While these polysaccharides and their isolation and (enzymatic) modification are important for food processing, in this review, we focus on the processing of nonedible fractions of crops and woody biomass and on the processing of xyloglucans, xylans, and glucomannans in particular.

## Lignocellulosic biomass, an entangled composite network

**Figure 1:**
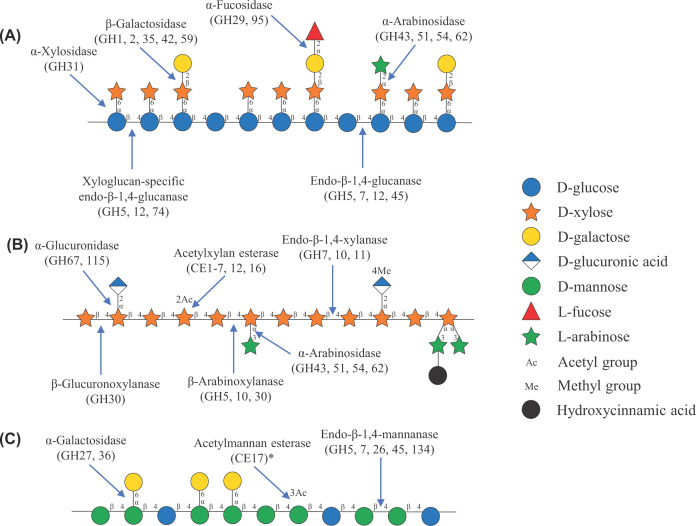
Structural representation of hemicellulosic polysaccharides and relevant hemicellulolytic enzyme activities Xyloglucan, acetylated glucuronoarabinoxylan, and acetylated galactoglucomannan are shown in panels (**A**–**C**), respectively. Symbol nomenclature was assigned according to the Symbol Nomenclature for Glycans (SNFG) [4,6]. The black circle represents hydroxycinnamic acid decoration, which occurs on 5-O of arabinosyl units of glucuronoarabinoxylan. Blue arrows and corresponding text illustrate the enzymatic activity that can contribute to depolymerization of the hemicellulosic polysaccharides. Primary CAZy families in which the enzymes are found are indicated in parentheses following the activity. The asterisk (*) assigned to the acetylmannan esterase activity indicates that activity may be found in other CE families with acetylxylan esterases, the activity of which has not been assessed previously on acetylated mannans.

**Figure 2:**
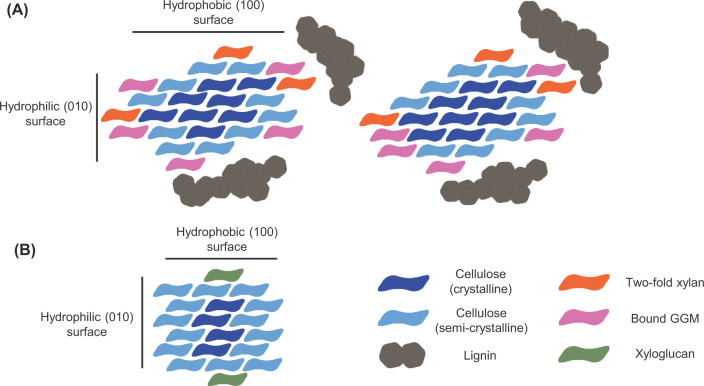
Binding of hemicellulosic polysaccharides to cellulose microfibrils Panel (**A**) shows a cross-section model of the binding of xylan and glucomannan to cellulose chains. In the figure, 2-fold screw xylan, shown in orange, and galactoglucomannan, shown in pink, are illustrated bound tightly to the hydrophilic surface of the cellulose microfibril (shown as cellulose chains in two shades of blue). Note that xylan and glucomannan can often adhere to the same cellulose microfibrils, and that the binding of these hemicellulosic polysaccharides slightly alters the structural conformation of the cellulose chains, illustrated by the difference in color of (some of) the cellulose polymers. (**B**) illustrates the binding of xyloglucan to the hydrophobic surfaces of a cellulose microfibril. The image shows a cross-section model of a cellulose microfibril containing 18 cellulose chains, with two bound xyloglucan moieties (shown in green). Panel (**A**) is reproduced based on the work by Terrett et al. [12]; panel (**B**) was created based on the results by Park et al. [9].

Regarding lignocellulose structure, it consists of a cellulose skeleton made up of cellulose fibrils which is embedded in a hemicellulose–lignin matrix. A detailed overview of the plant cell wall architecture is reviewed by Carpita and McCann [2]. Hemicellulose comprises various branched polysaccharides of diverse sugar molecules including xyloglucans, glucomannans, and xylans [3], as illustrated in Figure 1 (using the Symbol Nomenclature for Glycans (SNFG) [4,5, 6,7]). The peculiarity of hemicellulosic polysaccharides is their inhomogeneous nature; each of these polysaccharide types may contain segments with a particular frequency of substitutions that enables the backbone to align tightly to the surface of the cellulose microfibril when taking up a 2-fold helical screw, or flat ribbon-like, conformation (Figure 2). On the one hand, (XXXG)_*n*_-type xyloglucan (carrying only single xylosyl substitutions on the equatorial C6 hydroxyl groups) can strongly adhere to the hydrophobic (100) surface of the microfibril [8, 9,10]. On the other hand, xylans and glucomannans with evenly spaced substitutions (leaving one side of the polymer without substitutions and ready to interact with cellulose) bind tightly to the hydrophilic (010) surfaces of (often the same) cellulose microfibrils [11,12] (NB: Potential binding of xylan or glucomannan to the hydrophobic surface remains unknown). Such hemicelluloses are often termed as recalcitrant xyloglucan, xylan, or glucomannan as they are deeply embedded in the plant cell wall structure and are resistant to removal by (physico)chemical treatments.

In addition to coating cellulose microfibrils, hemicelluloses also interconnect the cellulose microfibril network and may also physically block access to cellulose microfibrils. As an example, arabinoxylan with varying degrees of acetylation can adhere to cellulose to varying extents [13]. Of note, in addition to the main hemicellulose types discussed above, plant cell walls also comprise mixed-linkage glucan [14,15], the role of which in plant cell wall remains to be elucidated [16,17]. Furthermore, hemicelluloses may also form covalent bonds with lignin in the cell wall, yielding phenyl glycosides, benzyl ethers, and γ-benzyl esters. These connecting structures, often termed lignin–carbohydrate complexes (LCCs), also contribute to lignocellulose recalcitrance [18]. The close association of hemicelluloses with plant cell wall polymers, including hydrogen bonds with cellulose microfibrils and covalent linkages with lignin, limits accessibility by enzymes and, to some extent, process chemicals, and ultimately also the ability to fractionate plant cell wall components.

## The fate of hemicellulose in lignocellulose biorefining

**Figure 3:**
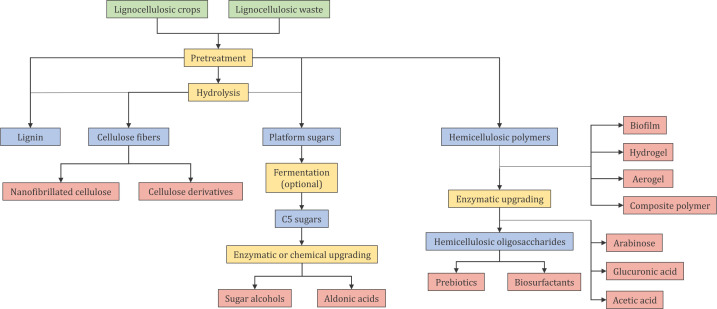
Potential main routes for the lignocellulose biorefinery, with a focus on hemicellulose valorization The scheme shows an array of value-added products (in red) that can be generated from hemicellulosic sugars and polymers, as well as from cellulose fibers. Processes are shown in yellow, while intermediate products are shown in blue.

Before lignocellulose can be processed efficiently into a variety of products (as exemplified in Figure 3), the tightly packed composite needs to be opened up by pretreatment. Pretreatment is the first step of biomass fractionation, leading to the (partial) separation of plant cell wall components [19]. The most common pretreatment technologies will, to some extent, remove and modify hemicellulose and/or lignin. Hemicellulose is dissolved and depolymerized to various extents, and can be recovered from the pretreatment liquor, depending on the pretreatment method, in a polymeric, oligomeric, and/or monomeric form. After an optional purification/separation step, hemicellulosic polymers, oligomers, and/or hemicellulosic sugars may be processed further for the generation of, for example, membranes, prebiotics, or chemicals such as xylitol (Figure 3). In addition, hemicelluloses remain, to some extent, in the solid cellulosic fraction in the form of loosely bound and recalcitrant hemicelluloses. Such hemicelluloses may be removed or modified in order to generate platform sugars by total saccharification or engineered cellulosic fibers, respectively (Figure 3).

From an economical perspective, the utilization and valorization of the complete biomass feedstock into various multifaceted production platforms is an essential goal for modern biorefineries, although many technical obstacles remain before this may become a reality. To maximize hemicellulose usability (including modification, removal, and/or recovery), either with the purpose of ‘refining’ cellulose or to valorize the hemicellulosic fraction, or both, a combination of hemicellulolytic enzymes must be utilized (see Table 1 for examples). The required enzyme activities will depend on innate properties of the feedstock (amount and types of hemicellulosic polysaccharides present), as well as on the pretreatment method utilized and on the targeted end product(s). In this review, we focus on the growing number of enzymatic bioprocessing technologies from the perspective of hemicellulose valorization. Hemicellulolytic enzyme activities required for processing lignocellulose as well as the enzymatic processes for the valorization of hemicellulose-containing biomass fractions are reviewed below.

**Table 1:** Applications of hemicellulolytic enzymes in hemicellulose valorization

Process	Product	Enzyme type	Status of technology	Reference
Total saccharification of lignocellulosic biomass	Platform sugars	Hemicellulase cocktail	Application at industrial scale	[20]
		LPMOs (Hemicellulolytic AA9)	Present in industrial cellulase cocktails; impact not assessed directly	Not yet reported
		Glucuronoyl esterases (CE18)	Developmental	[21]
		Endomannanases (GH5, GH26)	Developmental	[22]
Total saccharification of isolated hemicelluloses	C5 (pentose) sugars	Hemicellulase cocktail	Established; currently non-enzymatic industrially	[23]
	Mannose	(Gluco)mannanolytic enzyme cocktail	Product under development; (non-enzymatic) isolation from pretreatment liquor at pilot scale	[24,25]
Fiber engineering, hemicellulose removal	High-purity dissolving-grade pulp	Endoxylanases (GH10, GH11)	Developmental	[26,27]
		Endomannanases	Developmental	[26,28]
Fiber engineering, biobleaching by hemicellulose removal	Bleached paper pulp	Endoxylanases (GH10, GH11)	Application at industrial scale	[29]
		Endomannanases (GH5, GH26)	Established	[30,31]
		Debranching enzymes (α-galactosidase)	Developmental	[32]
Fiber engineering, increasing pulp yield	(Mechanical) pulp	Acetylmannan esterase	Developmental	[33]
Fiber engineering, cellulose fibrillation	Nanofibrillated cellulose	Endoxylanases (GH10, GH11)	Developmental	[34,35]
		LPMOs (AA9)	Developmental	[36]
		Endomannanases (GH26)	Developmental	[37]
Fiber engineering, limited hydrolysis	Cellulose nanocrystals	Endoxylanases, Endomannanases, LPMOs	Potential not yet explored	Not yet reported
Hemicellulose polymer engineering	Hydrogels	α-Glucuronidase (GH115)	Developmental	[38]
		α-Arabinosidase (GH54)	Developmental	[38]
		LPMOs (Hemicellulolytic AA9)	Potential not yet explored	Not yet reported
	Aerogels	Galactose oxidase (AA5)	Developmental	[39]
	Membranes, films	Arabinosidase	Developmental	[40]
	Hemicellulose-cellulose copolymers	Galactose oxidase (AA5)	Developmental	[41]
Limited hydrolysis of hemicellulose	Xylo-oligosaccharides	Endoxylanases (GH10, GH11, GH30)	Established; chemically by autohydrolysis of xylan-rich materials industrially	[42,43]
	Manno-oligosaccharides	Endomannanases (GH5, GH26, GH113, GH134)	Developmental; currently produced from yeast cell wall with autolysis industrially	[44]
	Xyloglucan oligosaccharides	Xyloglucanases	Developmental	[45]
Functionalization of hemicellulosic oligosaccharides (e.g., for derivatization to biosurfactants)	Ester-linked surfactants	Esterases (CE8), with acyltransferase activity	Developmental	[46]
	Alkyl glycosides	Endomannanases (GH5) and endoxylanases, with retaining mechanism	Developmental	[46]
	Site-specific oxidation for generation of carbohydrates for click chemistry/further functionalization	LPMOs (AA9) and carbohydrate oxidases and dehydrogenases (AA3)	Developmental	[46]
Enzymatic engineering of hemicellulosic oligosaccharides	Arabinose	α-Arabinosidase (GH43, GH51, GH54, GH62)	Developmental	[47]
	Glucuronic acid	α-Glucuronidase (GH115)	Developmental	[47]
	Acetic acid	Acetyl esterases (CE1, CE3-6, CE15-16)	Developmental	[47]
Enzymatic conversion of hemicellulosic sugars	Xylitol	Xylose reductase (EC 1.1.1.21)	Developmental; chemically at industrial scale	[48]
	Xylonic acid	Xylose dehydrogenases (EC 1.1.1.175)	Developmental	[49]
		Glucose oxidase (AA3; EC 1.1.3.4)	Developmental	[50]

## Hemicellulolytic enzymes

The large variation in composition and substitution pattern of hemicellulosic polysaccharides results in the formation of various co-polymeric substructures upon their interaction with cellulose [51]. As a result, numerous types of enzymes are often (co-)secreted by lignocellulolytic organisms to tackle these substructures and thereby achieve maximal degradation of the plant biomass. Relevant enzyme activities are illustrated in Figure 1, alongside the hemicellulosic polysaccharides upon which they act. Carbohydrate-active enzymes are collectively grouped and classified based on their catalytic mechanism and amino acid sequence within the carbohydrate-active enzymes (CAZy) database (http://www.cazy.org). The CAZy database currently harbors five distinct enzyme classes (glycoside hydrolases, GHs; glycosyltransferases, GTs; polysaccharide lyases, PLs; carbohydrate esterases, CEs; and auxiliary activities, AAs) as well as one class of associated modules (carbohydrate-binding modules, CBMs) [52].

### Hydrolases in hemicellulose depolymerization

Glycoside hydrolases (GHs) able to cleave the backbone of hemicellulosic polysaccharides include endo-β-1,4-xylanases, endo-β-1,4-mannanases, and xyloglucan-specific endo-β-1,4-glucanases, or shortly xylanases, mannanases, and xyloglucanases, respectively. Xylanases and mannanases are capable of cleaving between two (non-substituted) xylosyl or mannosyl units, respectively. In addition, xyloglucanases and certain xylanases, such as arabinoxylanases and glucuronoxylanases, can tolerate, and in some cases may even require, substitution on one of the two sugar moieties forming the scissile bond. These endo-acting enzymes cleave xylan, (gluco)mannan, or xyloglucan backbone at positions with certain substitution patterns and, because of the irregularity of the side chains, generate corresponding oligosaccharides of varying length, while simultaneously creating new chain ends for exo-acting enzymes.

The many side chains that can be found attached to hemicellulose polysaccharides and attribute to their wide chemical variability are removed by so-called debranching enzymes. Depending on the type of the scissile bond, debranching enzymes are classified as GHs (acting on glycosidic bonds) and carbohydrate esterases (CEs; acting on ester bonds). The former group includes arabinosidases, galactosidases, xylosidases, and glucuronidases, while the latter group includes acetyl, glucuronoyl, feruloyl, and *p*-coumaroyl esterases. Removal of substituting groups can be critical for xylanases and mannanases. In particular, removal of acetyl, (methyl)glucuronoyl, and galactosyl substitutions of recalcitrant hemicellulosic polysaccharides [12,53] facilitates access to the underlying cellulosic fibers by cellulolytic enzymes for complete biomass conversion or cellulose fiber engineering likewise. Furthermore, specialized CEs such as feruloyl and *p*-coumaroyl esterases [54,55] as well as glucuronoyl esterases [56,57] are needed in order to uncouple hemicellulose (fragments) from the (residual) lignin fraction via the cleavage of ester bonds typically found between lignin and carbohydrate moieties in LCCs. The advantage of CEs in lignocellulose processing is likely two-fold, as they aid hemicellulose–lignin separation and simultaneously assist in exposing underlying cellulose by facilitating depolymerization of hemicellulose fractions.

In nature, but also in certain biotechnological applications, xylan-, (gluco)mannan-, and xyloglucan-acting enzymes act in concert on a variety of types of the respective polymers. Malgas et al. provide a detailed overview of the xylan and mannan structures in nature and of the co-operativity of xylanolytic [23] and mannanolytic enzymes [24]. Moreover, Zavyalov et al. provide a detailed overview of the occurrence of xyloglucan substitution patterns in plant genera and discusses the xyloglucan-active GH types [45], some of which are cellulose-active endoglucanases as discussed in the next section.

### Endoglucanases with hemicellulolytic activity

Apart from the hemicellulose-specific enzymes discussed above, many endoglucanases (EGs) have been identified to be ‘promiscuous’ in the sense that they are capable of cleaving hemicellulosic polysaccharides such as xyloglucan, xylan, and glucomannan (but also mixed-linkage glucan) in addition to cellulose. It is, however, important to distinguish between the ability of an enzyme to depolymerize a particular substrate and its ability to do so efficiently or with a high catalytic proficiency. Activity profiles against xyloglucan, xylans, and/or mannans have been reported for a broad selection of EGs belonging to the GH families 5, 6, 7, 9, 12, and 45 in the seminal work by Vlasenko and co-workers [58]. As an example, the four EGs of *Trichoderma reesei* (anamorph of *Hypocrea jecorina*), *Tr*Cel5A, *Tr*Cel7B, *Tr*Cel12A, and *Tr*Cel45A, have different specificities toward hemicelluloses. In addition to cellulose, all four EGs have been reported to cleave glucomannan to varying extents [59,60]. Activity on xyloglucan has been reported for *Tr*Cel12A [61]. Furthermore, *Tr*Cel7B has comparable (if not higher) activity on xylan than on cellulose [62], illustrating that bifunctionality of enzymes has served an important evolutionary role for lignocellulolytic organisms. Harboring such enzymes with a wider scope of substrate specificity may provide an organism with an advantage during degradation of complex natural substrates. Supplementation of minimal cellulase cocktails with certain ‘promiscuous’ enzymes such as *Tr*Cel7B or *Tr*Cel5A has also been observed to improve saccharification of lignocellulosic biomass [63,64]. Strikingly, both *Tr*Cel7B and *Tr*Cel5A are able to solubilize residual glucomannans (approximately 1%, w/w) from microcrystalline cellulose (Avicel) and thereby boost cellulose depolymerization [63], indicating that the substrate promiscuity of EGs facilitates access to hemicellulose-coated recalcitrant cellulose microfibrils. It is important to note that it may be difficult to determine an enzyme’s preferred substrate, or ‘true’ substrate specificity, when the enzyme is exposed to a co-polymeric substrate. In the case of EGs active on hemicellulose as an example, the enzyme will forcibly depolymerize the cellulose-bound hemicellulose first in order to gain access to the underlying cellulose. Although in this sense the enzyme will act on the hemicellulose first, this does not necessarily imply that this is a better-suited substrate.

### Oxidative hemicellulose-active enzymes

Lignocellulose-degrading organisms also utilize oxidative enzymes such as lytic polysaccharide monooxygenases (LPMOs) to depolymerize hemicellulosic polysaccharides. These enzymes play an important role in lignocellulose degradation because similarly to endo-acting GHs, LPMOs create novel chains ends for exo-acting enzymes. Plant cell wall-active LPMOs belong to the Auxiliary Activity (AA) families 9, 10, 16, and 17. Of these, activity on plant cell wall polysaccharides has been observed on cellulose for AA9s, AA10s, and AA16s, and on pectin for AA17s. For a number of AA9 LPMOs, convincing activity on hemicelluloses has also been shown in addition to activity on cellulose [65,66] (of note, activity on cellulose-bound recalcitrant xylan, and thus on plant cell walls, by AA14s has been reported for one AA14 protein only in the original report [67] and is currently debated in the field). Following initial discoveries of LPMO activity on chitin [68] and cellulose [69–73], the first LPMO activity on hemicellulose was observed in 2014 [74]. Agger and colleagues demonstrated activity of *Nc*LPMO9C from *Neurospora crassa* on several (isolated) hemicellulosic polysaccharides, including xyloglucan, glucomannan, and mixed-linkage glucan. Shortly thereafter, the first LPMO with catalytic activity on cellulose-associated xylan was reported in 2015 by Frommhagen and colleagues [75]. Since then, several more LPMOs with hemicellulolytic activity have been identified [65,76–78]. Of note, all LPMOs hitherto found to cleave cellulose-associated xylan (or xyloglucan) are also active on cellulose, albeit to somewhat varying extents (as discussed in [78]). For two xylan-active LPMOs, *Nc*LPMO9F from *N. crassa* and *Tt*LPMO9E from *Thermothielavioides* (prev. *Thielavia*) *terrestris*, (cellulose-associated) xylan depolymerization capability appears to be stronger than their activity on cellulose, and perhaps more biologically relevant [77,78]. Importantly, some of the xyloglucan-active but all of the xylan-active LPMOs have been found active only on hemicellulose associated with cellulose. This phenomenon has been attributed to the difference in the conformation of the free (threefold helical screw) and cellulose-bound (twofold helical screw) polymers (see Figure 2) and their potential interaction with the flat substrate-binding surface of LPMOs.

The Auxiliary Activities enzyme class also contains enzymes that oxidize hemicellulose-derived poly-, oligo-, and monosaccharides. AA5_2 galactose oxidases can oxidize the C6-hydroxyl of terminal (nonreducing) galactosyl substitutions in xyloglucan and galactoglucomannan [79]. Nonspecific AA3_1 cellobiose dehydrogenases [78,80,81] or AA7 gluco-oligosaccharide oxidases [82] can oxidize non-substituted reducing-end sugars in xyloglucan-, glucomannan-, and/or xylan-derived oligosaccharides. Hemicellulose-specific oxidases, such as xylooligosaccharide oxidase (AA7), can oxidize the terminal non-substituted reducing-end sugar in specific oligosaccharides [83]. Although these enzymes do not per se depolymerize hemicelluloses, they contribute to value creation from biomass by functionalization of hemicellulose-derived biomaterials.

## Hemicellulolytic enzymes in lignocellulose processing

In general, hemicellulose-active enzymes can be used for two main purposes in lignocellulose processing: (1) hemicellulose removal to clean cellulose fibers and increase accessibility of cellulose microfibrils for total saccharification or fiber engineering in lignocellulose biorefineries; (2) conversion of isolated hemicellulose fractions to distinct streams of poly-, oligo-, or monomeric products (or intermediates). These processes are illustrated within Figure 3, which shows an overview of the main routes within the lignocellulose biorefinery with a focus on the fate and potential of hemicellulose.

Some of these aspects have recently been reviewed by [84,85]. In this section, we summarize the existing and potential applications of hemicellulolytic enzymes, which are also summarized in Table 1.

### Complete saccharification of lignocellulosic biomass

One of the most explored and industrially implemented conversion routes of lignocellulosic biomass involves the complete depolymerization of polysaccharides remaining in the biomass after pretreatment to platform sugars (including glucose and other sugar monomers) and the fermentation thereof to a variety of chemicals, the main being ethanol [86]. The majority of the feedstocks include agricultural residues and energy crops, both rich in xylan, while utilization of forest residues, rich in both xylan and glucomannan, is also substantial [87]. Importantly, concomitant depolymerization of cellulose and hemicellulose is critical in order to achieve complete saccharification of lignocellulosic biomass [63,88]. Depending on the type of pretreatment method that the biomass has been subjected to, the feedstock will vary in hemicellulose content [89]. Xylanases are currently incorporated into major commercially available cellulase preparations, such as Novozymes' Cellic CTec2 [90] and DuPont's Accellerase Trio [91,92]. However, for the saccharification of xylan-rich feedstocks, hemicellulolytic enzyme cocktails (e.g., Novozymes' Cellic HTec and DuPont's Accellerase XC) are also commercially available to be used in combination with cellulase cocktails for the degradation of hemicellulosic polysaccharides while simultaneously facilitating cellulose hydrolysis. The importance of endo-active and debranching hemicellulose-active enzymes in complete saccharification of lignocellulosic biomass has been recently reviewed [85,20]. Specific examples include the use of endomannanases [22,93] and glucuronoyl esterases [21,94] to improve biomass saccharification. It is noteworthy that promiscuous EGs, which may be key in enabling cellulose accessibility by removing recalcitrant hemicelluloses coating cellulose microfibrils, are naturally present in fungal secretomes and therefore in commercial cellulase cocktails.

Interestingly, some of the LPMOs performing best in industrially relevant setups [95] are promiscuous LPMOs; *Ta*LPMO9A from *Thermoascus aurantiacus* is active on cellulose and xyloglucan [96], while *Tt*LPMO9E from *T. terrestris* is active on cellulose and xylan [77]. While contributions of cellulose-active LPMOs to biorefining by way of addition to commercial cellulase cocktails is well established [20,97–99], the potential and effective contribution of hemicellulolytic LPMOs to lignocellulose bioprocessing remain relatively underexplored.

### Cellulosic fiber engineering

Cellulosic fibers can be processed into commodity pulp for paper production or into dissolving pulp to produce textile fibers (such as viscose) or specialty cellulose (such as cellulose derivatives or nanocellulose) [84]. One of the first examples of enzymatic cellulose fiber engineering dates back to the late 1980s when xylanases were implemented in order to partially replace chlorine-based bleaching agents [29,100]. In fact, endo-acting xylanases and mannanases [101, 31,102] and debranching enzymes like α-galactosidase [32], but also lignin-active oxidoreductases like laccase [103], have been shown to improve lignin removal and thereby increase pulp brightness. Enzymes with potential in bleaching chemical pulp have been reviewed recently by Immerzeel and Fiskari [104]. The mechanisms behind increasing pulp brightness are attributed to removal of reprecipitated xylan, hexenuronic acid (a degradation product of glucuronic acid in glucuronoxylan), and LCCs (with covalently bound lignin moieties) and increase in fiber permeability of bleaching chemicals.

Paper-grade wood pulp can be upgraded to dissolving pulps by lowering hemicellulose (and lignin) content. Xylanases have been explored successfully for the co-production of dissolving-grade pulp and xylan from paper-grade wood pulp [105,26]. Furthermore, xylanases and mannanases have been shown to decrease hemicellulose content from dissolving pulp [28, 27,106], thereby increasing its value. Complete removal of the residual (structural) hemicelluloses, a prerequisite for the production of cellulose derivatives, is, to date, achieved chemically [107], while enzymes have been shown to enhance hemicellulose removal from paper-grade pulp when combined with hot caustic extraction [30].

Nano- and microfibrillated cellulose (NFC and MFC, respectively) [108] as well as cellulose nanocrystals (CNCs) [109] are three of the new value-added products from cellulose fibers where enzyme applications have been gaining increasing attention. Treatment of the cellulose pulp with xylanase, mannanase, EG, and/or LPMO prior to the mechanical refining step has been reported to promote the formation of a fibrillar network [31, 33, 38, 103,34, 36, 41, 110, 35, 111, 37,112]. Implementing enzymatic treatment simultaneously with refining has been suggested to intensify enzyme treatment [113]. A recently reported process configuration allows generation of MFCs/NFCs (which are strong hydrogels with very low, typically 1–3%, w/w, solid content) at very high solid content (25%, w/w) with simultaneous enzymatic and mechanical treatment [114,115]. Removal of hemicellulose, xylan in particular, facilitates initial stage fibrillation, leading to energy reduction during subsequent mechanical refining [36,34]. On the other hand, removal of glucuronoxylan also decreases colloidal stability due to a decrease in repulsive force that hinders fiber aggregation, as a result of removal of carboxyl groups [116]. The need for and extent of specific hemicellulose removal by enzymes will depend on the application and desired material properties of the hydrogel. Nanofibril suspensions may be stabilized by fiber treatment with a C1- or C1/C4-oxidizing LPMO (*Nc*LPMO9F from *N. crassa* [117] and *Ta*LPMO9A from *T. aurantiacus* [36], respectively) which introduces carboxylic groups (at C1 position) on cellulose, and perhaps also on xylan (for *Nc*LPMO9F [78]) or xyloglucan (for *Ta*LPMO9A [96]). Of note, the hemicellulolytic side activity of EGs and LPMOs has not been considered so far, and their impact on the hemicellulose fraction of the pulp remains to be elucidated. Carboxylic groups, which allow chemical functionalization of MFCs/NFCs in downstream processes, are routinely generated by laccase/TEMPO oxidation [118]. Alternatively, AA5_2 galactose oxidases could be used to oxidize the C6-hydroxyl of terminal (nonreducing) galactosyl substitutions in residual xyloglucan and galactoglucomannan fractions [79]. It is noteworthy that xyloglucan-cleaving enzymes (such as *Tr*Cel7B or *Ta*LPMO9A) may play an important role in fiber processing in addition to xylan- and glucomannan-active enzymes, primarily because they could contribute to the removal of primary cell wall moieties from cellulosic fibers by breaking xyloglucan crosslinks between cellulose fibrils and thereby enhance fiber swelling and solubilization. Although primary cell wall constitutes less than approximately 5% (v/v) of the wood cell wall [119], removing primary cell wall moieties from the fiber surface has a remarkable impact on fiber swelling and dissolution, as demonstrated with chemical means by, for example, Trygg and Fardim [120].

Cellulose nanocrystals (CNCs) are most commonly produced via acid hydrolysis of nonfibrillated or fibrillated cellulose fibers [109,121]. In some cases, enzymatic production of CNCs (with EGs and without an additional acid hydrolysis step) has also been reported, yielding CNC with limited colloidal stability and uniformity and low crystallinity [122]. In a biorefinery concept, CNCs may be isolated from the solid saccharification residues remaining after an incomplete saccharification step (i.e., breaking down amorphous cellulose while retaining the more recalcitrant crystalline cellulose), either by filtration [123] or by acid hydrolysis [124]. A recent study reports that isolating CNCs (by centrifugation and sonication) from the saccharification residue of bleached eucalyptus kraft pulp yields CNCs with properties (regarding thermal stability, crystallinity index, and particle diameter uniformity) superior to CNCs produced by acid hydrolysis from the nonhydrolyzed biomass [125]. CNCs isolated by acid hydrolysis are often considered pure cellulose nanocrystals (even if the chemical composition of CNCs varies significantly depending on the isolation method [126]), whereas CNCs isolated enzymatically are expected to contain considerable amounts of (recalcitrant) hemicellulose. On the one hand, hemicellulose content may lower crystallinity; on the other hand, glucuronic acid side groups may improve colloidal stability. The impact of hemicellulose on CNC properties is yet to be determined. Thus, hemicellulases may be used together with EGs or cellulases to break down the amorphous parts of the biomass and generate a saccharification residue with higher crystallinity and particle diameter uniformity, or even to alter residual (e.g., recalcitrant) hemicelluloses covering CNCs.

### Enzymatic upgrading of hemicellulosic polymers

In a lignocellulose biorefinery, hemicellulose polymers can be shaped into membranes, coating films, hydrogels, and aerogels—either alone or when reinforced with, for example, cellulosic materials such as nanofibrillated cellulose [127,128]. For that, hemicelluloses may be isolated in a polymeric form with a relatively well-defined length during pretreatment, for example, when using hot water extraction [129]. The extraction method, i.e., the type of pretreatment, will determine the structure, as severe conditions can cause dehydration, oxidation, or hydrolysis of sensitive sugars as well as deacetylation of the polymer [127,128]. The substitution pattern of the polymer impacts the physico-chemical properties of the resulting membranes and 3D structures, including mechanical strength, flexibility, thermal behavior, and oxygen or water barrier properties [130,131]. In addition to the type of extraction method and chemical modifications, physico-chemical properties can be tailored by enzymatic processes, which include (1) selective removal of substitutions, (2) oxidation of selected substitutions, and (3) cross-linking (reviewed also by [127,132,47]). Interestingly, the released side groups, especially (methyl)glucuronic acid from xylan, may be recovered after enzyme treatment as a co-product, as discussed recently by Vuong and Master [47].

Enzymatic debranching of hemicelluloses, including removal of acetyl, arabinosyl, and glucuronoyl substitutions, alter not only physico-chemical but also biological properties, and thus have been explored for a range of applications over the past decades, from increasing pulp yield [33] to health applications [133,134]. Deacetylation in general decreases polymer solubility in aqueous solutions [33,135]. In the case of xylans, removing (1→2)- and/or (1→3)-linked arabinosylations by arabinosidases decreases the solubility of the polymer in water and yields films with increased degree of crystallinity, improved mechanical properties, and lower oxygen permeability [40]. Moreover, removing arabinosylations to create xylans with various substitution patterns promotes association of xylan with cellulose [13,136]). Removal of glucuronoyl substitutions promotes xylan precipitation in hydrogel production both without [38] or with a reinforcing cellulosic material [137].

Galactosyl substitutions in glucomannans and xyloglucans, on the other hand, are primarily targeted for functionalization. Selective oxidation of (terminal) galactosyl substitutions with galactose oxidase creates an aldehyde group at the C_6_ carbon in glucomannan and xyloglucan [138], which is a hotspot for (chemical) functionalization [139] and can facilitate interaction with other (co)polymers [140]. Xyloglucan has been shown to form a hydrogel after galactose oxidase treatment [79]. In addition to hemiacetal crosslinking via the galactoaldehyde functionality for hydrogel and aerogel production [79,39], the laccase/TEMPO system and peroxidases have also been used to produce aldehyde derivatives of the sugar building blocks for hemiacetal-crosslinked hydrogels and aerogels [141] or to directly crosslink hydroxycinnamoyl substitutions in xylans [47]. Furthermore, hemicellulose-active AA9 LPMOs can potentially be used to introduce one or two functional groups at one (or both) of the chain ends (C_1_ carboxylic and/or C_4_ keto (or, in aqueous medium, gem-diol) groups at the reducing and nonreducing ends, respectively) when cleaving xyloglucan, glucomannan, or xylan. While some AA9 LPMOs can cleave xyloglucan or glucomannan in solution [74], activity on xylan has only been found for xylans bound on cellulose [75,77,78]. LPMO functionalization of hemicelluloses, however, remains to be explored in carbohydrate polymer chemistry.

### Production and upgrading of hemicellulosic oligosaccharides

In addition to hemicellulosic polymers, hemicellulosic oligosaccharides have been explored for a wide range of applications, for example, as prebiotics for human consumption or in animal feed [142,44], plant elicitors in agriculture [143], and biosurfactants [46], in order to further diversify the portfolio of a lignocellulosic biorefinery. Xylo- and manno-oligosaccharides can be generated by limited hydrolysis of the corresponding hemicellulosic polymers, using e.g. chemical or enzymatic hydrolysis, or direct autohydrolysis [142,44,42,43,144,145]. Due to its high degree of specificity, rather uniform product profile, and minimal production of by-products, enzymatic hydrolysis (using xylanases, β-xylosidases, and mannanases) may be the best-suited method for production of oligosaccharides especially for prebiotic or medicinal use.

The bioactivity (such as fermentability by gut microbiota and antioxidant properties) of hemicellulosic oligosaccharides is dependent on the substituting groups; therefore, debranching enzymes, including acetylases and glucuronidases, are important tools in tailoring the oligosaccharides. As an example, non-substituted and arabinosylated xylo-oligosaccharides are fermented more quickly than acetylated and methylglucuronoylated xylo-oligosaccharides and hence may be more suitable for promoting growth of *Bifidobacteria* [146,147] or human fecal inocula [148]. On the other hand, methylglucuronoylations seem to provide antioxidant properties to xylo-oligosaccharides [149]. Similarly to acetylated spruce arabinoglucuronoxylan-derived oligosaccharides, acetylated spruce galactoglucomannan-derived oligosaccharides have been shown to increase the probiotic *Bifidobacterium* population in human fecal inocula [150]. The sidechains of hemicellulosic oligosaccharides are also important in their role in plant signaling. Xyloglucan-derived oligosaccharides carrying fucosyl-galactosylation (such as in the nonasaccharide XXFG) have been shown to inhibit plant growth [143], while galactosylation influences the biological activity of galactoglucomannan-derived oligosaccharides [151].

Hemicellulosic oligosaccharides can also be derivatized to generate a variety of glycoconjugates which can serve as platform chemicals with a range of further applications [152]. While sugar-based biosurfactants are traditionally produced via derivatization of the aldehyde group of the reducing-end sugar using synthetic methods such as click chemistry [152], enzymatic processes have been explored for linking mono- or oligosaccharides to long aliphatic chains at alternate positions, as recently reviewed in detail by Agger and Zeuner [46]. As an example, oligosaccharides may be coupled to long-chain fatty acids via transamination, after reductive amination of the reducing-end aldehyde [153], or directly via stereoselective enzymatic esterification of, for example, the C_4_ hydroxyl of the nonreducing-end sugar by a lipase [154]. In the first step of derivatization, the aldehyde group may be oxidized to a carboxylic group at the reducing end by hemicellulose-specific reductases and oxidases. The aldehyde group of a nonsubstituted glucose or xylose unit located at the reducing end of the hemicellulosic oligosaccharide backbone have been reported to serve as substrates for AA3 cellobiose dehydrogenase [78,80,81] or AA7 gluco-oligosaccharide oxidase [82]. Furthermore, dehydrogenases and oxidases leading to oxidation at the C_2_ or C_6_ carbon [155] result in opportunities for derivatization of hemicelluloses at positions other than the non-reducing end, which is difficult to achieve using synthetic means. In fact, hemicellulosic oligosaccharides may be functionalized at the non-reducing end already during limited saccharification of the polymer by C_4_-oxidizing LPMOs, as exemplified by a recent report on the generation of hemicellulosic glycoconjugates with the C_4_-oxidizing *Nc*LPMO9A [156].

### Production and valorization of hemicellulosic sugars as platform sugars

The benefits of the use of hemicellulases in lignocellulose processing extend beyond increased facilitation of cellulose hydrolysis, as the specific action of various hemicellulolytic enzymes will result in the generation of a mix of hemicellulosic sugars that may serve as platform chemicals [157,158]. Depending on the pretreatment, hemicellulosic sugars may be recovered directly (or after depolymerization with acid or hemicellulases) from the pretreatment liquor or from the enzymatic hydrolysate [19]. Alternatively, pentoses (xylose and arabinose) may be recovered after selective fermentation of the hexoses (primarily glucose and mannose) solubilized during the saccharification step, for example, with wild-type *S. cerevisiae* strains, which cannot catabolize and ferment pentoses [159]. Strain engineering has led to the development of yeast strains co-fermenting hexoses (C6) and pentoses (C5 sugars) to ethanol [94]; one of the engineered strains being Novozymes' commercially available Cellerity® 1.0 yeast strain.

Most commonly, hemicellulases may be used to monomerize partially depolymerized hemicelluloses in the pretreatment liquor or hemicelluloses remaining in the fiber fraction. Moreover, hemicellulose-active redox enzymes may be used to increase the value of pentose sugars by converting them to alditols (such as xylitol and arabinitol, two among the top twelve value-added chemicals from biomass [93]) or aldonic acids (such as xylonic acid [49, 50,160, 161]). Both sugar alcohols and sugar acids can be produced by chemical, microbial, or enzymatic (using specific sugar dehydrogenases) reduction [48] or oxidation [162], respectively. However, reduction of pentose sugars, such as xylose to xylitol, is currently done chemically at industrial scale, one example being DuPont’s trademark process producing XIVIA Xylitol from xylose remaining after pulp cooking of corn stover [106].

### Process parameters and competitiveness of enzymatic processes at industrial scale

Process parameters such as substrate loading (dry matter content), mixing, control of temperature and pH, and the type (and price) of reactants determine the economic viability of the industrial processing of lignocellulose. Some of these factors largely depend on intrinsic properties of the substrates utilized (e.g., maximum possible substrate loading), while others could, in some cases, be tailored to suit the specific properties of enzymes (e.g., pH and temperature stability and optima) when designing enzymatic processes. Notably, there is less flexibility to tailor process parameters in already established processes, such as industrial bleaching of pulps, in comparison with technologies that are developed around the enzymatic catalysis itself, such as total saccharification of biomass to platform sugars. With the continuous expansion of databases, enzymes with industrial potential are nearly limitless. In addition to traditional strategies for enzyme improvement [102], a rapidly expanding number of whole genome sequences provide an enormous source of proteins by functional metagenomics [111] for identification of enzymes with industrial potential. In the case of total saccharification, concentrating on fungal plant cell wall-active enzymes, which are compatible with process pH (4.5–5.0) and temperature (45–50°C), helps focus the efforts.

To identify enzymes with properties (such as affinity, stability, and reduced end-product inhibition) suitable for industrial processes, enzymes need to be screened under industrial conditions. One of the best examples comes from cellulases, regarding enzyme modularity. It is now well-established that the presence of a carbohydrate-binding module (CBM), a non-catalytic domain of bi- or multi-modular GHs that assists in targeting the catalytic domain of the enzyme to the appropriate region of the substrate [112], is of less importance to a GH when operating under higher dry matter conditions [144,145]. The minimized contribution of a CBM to GH activity at higher dry matter content can be explained by the more frequent occurrence of enzyme–substrate interactions at higher dry matter concentrations, and due to the faster off-rates of GHs lacking CBMs, resulting in less unproductive binding of the enzymes to the substrate. However, this phenomenon may not apply to CBMs that are pivotal to substrate recognition and catalytic action [112].

Higher dry matter content (and thus lower water content) is generally preferable in lignocellulose processing under industrial conditions as this will result in higher sugar and alternate end product yields, lower energy costs associated with temperature regulation of the reactor, and minimization of waste water [160,161]. For the majority of lignocellulosic feedstocks, the substrate loading must be equal to 15% (w/w) dry matter or higher for the process to be considered economically viable (from the perspective of ethanol production) [160]. However, operation of bioreactors with such high solid loadings also poses significant practical challenges, for example, associated with adequate mixing (i.e., inadequate mixing due to high viscosity of high dry matter-containing slurries, or high energy costs associated with adequate mixing) or with corresponding high production of end products and inhibitory molecules, which may reduce enzyme activity [161,5]. Enzymatic processes at high dry matter have been established for total saccharification of biomass to platform sugars [7] and pulp bleaching [29] — processes implemented at industrial scale (see also Table 1). On the other hand, some processes, such as aerogel production, inherently require low (typically 1–2%, w/v) substrate loading [39,141]. Other processes, such as enzymatic (nano)fibrillation of cellulose, have been successfully developed at higher (20–40%) substrate loading from dilute systems (1–3%) [10], thereby creating a new type of material.[94, 10] While technology development is enabling the (co-)production of products with established markets, for new products (such as mannose [25,162]), market identification and creation must be pursued before commercial-scale production and value generation can begin.

When evaluating enzymatic processes in comparison to competing (physico)chemical technologies, process economy is the determining factor for practical feasibility. On the one hand, enzyme instability, longer residence time, and the high cost of enzymes (i.e., compared with that of bulk chemicals) may decrease competitiveness of enzymatic processes. Accordingly, low enzyme doses and short residence times are desired process parameters. On the other hand, high selectivity, simplicity, high conversion efficiency of the reaction, or more uniform products can boost competitiveness, and a novel type of catalysis or the lack of toxic reagents or by-products can create new markets for enzymatic processes (e.g. in food applications).

## Summary

- Increasing oil prices and the heavy environmental burden of crude oil are resulting in increased global attention to more sustainable solutions such as lignocellulose biorefineries.
- The economic viability and competitiveness of the lignocellulose biorefinery requires complete utilization of local lignocellulosic resources and diversification of the product portfolio, for example, by complementing low-value energy carriers (such as ethanol) with high-value products (such as fibrillated cellulose, hemicellulose-based prebiotics, and lignin-derived specialty chemicals).
- Hemicellulose has a high potential to be utilized in polymeric (e.g., for membranes and hydrogels), oligomeric (e.g., for prebiotics and biosurfactants), and monomeric forms (as platform chemicals), and is underutilized in current biorefinery processes.
- The use of enzymatic processes (which are highly selective and thus capable of fine-tuning (hemi)celluloses) has seen an acceleration over the past decades, and these processes are expected to be preferred to chemical processes in the future circular bioeconomy, especially for the production of hemicellulosic polymers and oligomers for the healthcare (prebiotics or drug delivery agents) and food (packaging or food additives) industries.
- Hemicellulose-active enzymes are powerful tools not only for adding value to the currently underutilized hemicellulose fraction of lignocellulose, but also for upgrading cellulosic products, as highlighted in this review.

## Abbreviations

AA: auxiliary activity
CAZy: carbohydrate-active enzymes
CBM: carbohydrate-binding module
CE: carbohydrate esterase
CNC: cellulose nanocrystal
EG: endoglucanase
GH: glycoside hydrolase
LCC: lignin–carbohydrate complex
LPMO: lytic polysaccharide monooxygenase
MFC: microfibrillated cellulose
NFC: nanofibrillated cellulose
